# {8-[4-(Bromo­meth­yl)benzo­yl]-2,7-dimeth­oxy­naphthalen-1-yl}[4-(bromo­meth­yl)phen­yl]methanone

**DOI:** 10.1107/S1600536811029151

**Published:** 2011-07-23

**Authors:** Kosuke Sasagawa, Daichi Hijikata, Akiko Okamoto, Hideaki Oike, Noriyuki Yonezawa

**Affiliations:** a Department of Organic and Polymer Materials Chemistry, Tokyo University of Agriculture & Technology, Koganei, Tokyo 184-8588, Japan

## Abstract

In the title compound, C_28_H_22_Br_2_O_4_, the two 4-bromo­methyl­benzoyl groups at the 1- and 8-positions of the naphthalene ring system are aligned almost anti­parallel, the benzene rings forming a dihedral angle of 2.94 (16)°. The dihedral angles between the benzene rings and the naphthalene ring systems are 70.98 (13) and 72.89 (13)°. In the crystal, centrosymmetric­ally-related mol­ecules are linked into dimeric units by inter­molecular C—H⋯O inter­actions.

## Related literature

For formation reactions of aroylated naphthalene compounds *via* electrophilic aromatic substitution of naphthalene deriv­atives, see: Okamoto & Yonezawa (2009 ▶). For the structures of closely related compounds, see: Muto *et al.* (2010 ▶); Nakaema *et al.* (2007 ▶, 2008 ▶); Watanabe, Nagasawa *et al.* (2010 ▶); Watanabe, Nakaema *et al.* (2010 ▶).
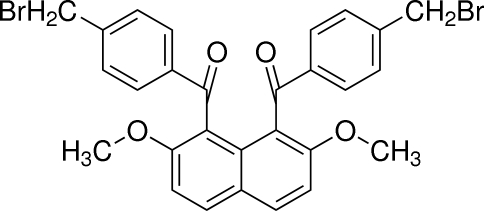


## Experimental

### 

#### Crystal data

C_28_H_22_Br_2_O_4_
*M* *_r_* = 582.28
Monoclinic, 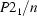

*a* = 11.5948 (2) Å
*b* = 8.37239 (15) Å
*c* = 24.5352 (5) Å
β = 92.617 (1)°
*V* = 2379.29 (8) Å^3^
*Z* = 4
Cu *K*α radiation
μ = 4.60 mm^−1^
*T* = 193 K
0.50 × 0.40 × 0.20 mm

#### Data collection

Rigaku R-AXIS RAPID diffractometer
Absorption correction: numerical (*NUMABS*; Higashi, 1999 ▶) *T*_min_ = 0.207, *T*_max_ = 0.460
41450 measured reflections
4319 independent reflections
3916 reflections with *I* > 2σ(*I*)
*R*_int_ = 0.079

#### Refinement

*R*[*F* ^2^ > 2σ(*F* ^2^)] = 0.041
*wR*(*F* ^2^) = 0.103
*S* = 1.14
4319 reflections
310 parameters
H-atom parameters constrained
Δρ_max_ = 0.85 e Å^−3^
Δρ_min_ = −0.59 e Å^−3^

### 

Data collection: *PROCESS-AUTO* (Rigaku, 1998 ▶); cell refinement: *PROCESS-AUTO*; data reduction: *CrystalStructure* (Rigaku/MSC, 2004 ▶); program(s) used to solve structure: *SIR2004* (Burla *et al.*, 2005 ▶); program(s) used to refine structure: *SHELXL97* (Sheldrick, 2008 ▶); molecular graphics: *ORTEPIII* (Burnett & Johnson, 1996 ▶); software used to prepare material for publication: *SHELXL97*.

## Supplementary Material

Crystal structure: contains datablock(s) I, global. DOI: 10.1107/S1600536811029151/rz2629sup1.cif

Structure factors: contains datablock(s) I. DOI: 10.1107/S1600536811029151/rz2629Isup2.hkl

Supplementary material file. DOI: 10.1107/S1600536811029151/rz2629Isup3.cml

Additional supplementary materials:  crystallographic information; 3D view; checkCIF report

## Figures and Tables

**Table 1:** Hydrogen-bond geometry (Å, °)

*D*—H⋯*A*	*D*—H	H⋯*A*	*D*⋯*A*	*D*—H⋯*A*
C17—H17⋯O1^i^	0.95	2.55	3.417 (4)	152
C28—H28*B*⋯O3^i^	0.99	2.50	3.453 (4)	162

Symmetry code: (i) 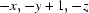.

**Fig. 1.**
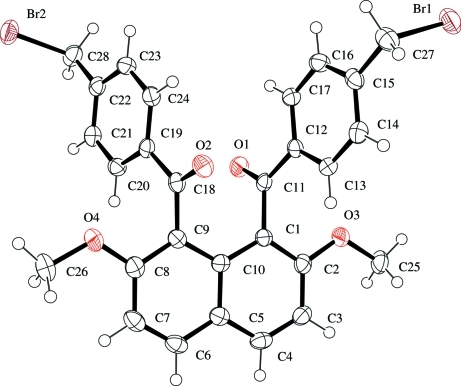
The molecular structure of title compound. Displacement ellipsoids are drawn at the 50% probability level.

**Fig. 2.**
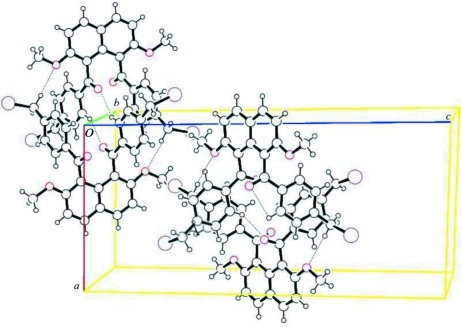
Partial crystal packing of the title compound, showing the intermolecular C—H···O interactions as dashed lines.

## supplementary crystallographic information

### Comment

In the course of our study on selective electrophilic aromatic aroylation of the naphthalene core, 1,8-diaroylnaphthalene compounds have proved to be formed regioselectively by the aid of a suitable acidic mediator (Okamoto & Yonezawa, 2009). Recently, we reported the X-ray crystal structures of 1,8-diaroylated 2,7-dimethoxynaphthalene derivatives such as 1,8-bis(4-chlorobenzoyl)-2,7-dimethoxynaphthalene (Nakaema *et al.*, 2007), 1,8-dibenzoyl-2,7-dimethoxynaphthalene (Nakaema *et al.*, 2008), bis(4-bromophenyl)(2,7-dimethoxynaphthalene-1,8-diyl)dimethanone (Watanabe, Nakaema *et al.*, 2010), (2,7-dimethoxynaphthalene-1,8-diyl)bis(4-fluorophenyl)dimethanone [1,8-bis(4-fluorobenzoyl)-2,7-dimethoxynaphthalene] (Watanabe, Nagasawa *et al.*, 2010), and 1,8-bis(4-methylbenzoyl)-2,7-dimethoxynaphthalene (Muto *et al.*, 2010). The aroyl groups in these compounds are attached to the naphthalene rings in an almost parallel fashion and oriented in opposite direction. As a part of our ongoing studies on the molecular structures of homologous aroylated 2,7-dimethoxynaphthalene molecules, the X-ray crystal structure of the title compound, bis(4-bromomethylbenzoylated) 2,7-dimethoxynaphthalene, is discussed in this article.

The molecular structure of the title compound is displayed in Fig 1. The two 4-bromomethylbenzoyl groups are twisted away from the attaching naphthalene ring and situated in *anti* orientation. The dihedral angle between the planes of the benzene rings is 2.94 (16)°. On the other hand, the dihedral angles of the benzene rings with the naphthalene ring system are 70.98 (13) and 72.89 (13)°, respectively. The torsion angles between the carbonyl groups and the naphthalene ring [C10—C1—C11—O1 = -68.9 (4)° and C10—C9—C18—O2 = -67.8 (4)°] are larger than those between the carbonyl groups and the benzene rings [O1—C11—C12—C13 = -177.3 (3)° and O2—C18—C19—C20 = -176.1 (3)°].

In the crystal packing, C—H···O hydrogen bonds between the oxygen atoms of the carbonyl groups and the hydrogen atoms of the benzene rings and between the oxygen atoms of the methoxy groups and the hydrogen atoms of the bromomethyl groups are also observed (Table 1), resulting in the formation of supramolecular dimeric units (Fig. 2) having crystallographic inversion centre.

### Experimental

To a 50 ml flask, 4-bromomethylbenzoic acid (22.0 mmol, 4.73 g) and phosphorus pentoxide–methanesulfonic acid (P_2_O_5_–MsOH, 40.0 ml) were placed and stirred at 333 K. To the mixture thus obtained, 2,7-dimethoxynaphthalene (10.0 mmol, 1.88 g) was added. After the reaction mixture was stirred at 333 K for 1 h, it was poured into ice-cold water (30 ml) and the mixture was extracted with CHCl_3_ (15 ml × 3). The combined extracts were washed with 2 *M* aqueous NaOH followed by washing with brine. The organic layers thus obtained were dried over anhydrous MgSO_4_. The solvent was removed under reduced pressure to give the crude product (81% yield), which was purified by reprecipitation from CHCl_3_-hexane (1:1 *v*/*v*; 57% isolated yield). The isolated product was crystallized from acetone to give single-crystal.

Spectroscopic Data:

^1^H NMR δ (300 MHz, CDCl_3_); 3.69 (6*H*, s), 4.49 (4*H*, s), 7.20 (2*H*, d, J = 9.0 Hz), 7.33 (4*H*, d, J = 8.0 Hz), 7.63 (4*H*, d, J = 8.0 Hz), 7.96 (2*H*, d, J = 9.0 Hz) p.p.m..

^13^C NMR δ (100 MHz, CDCl_3_); 32.7, 56.5, 111.4, 121.4, 125.6, 128.7, 129.5, 129.8, 132.2, 138.6, 141.9, 156.5, 196.1 p.p.m..

IR (KBr); 1666 (C=O), 1606 (Ar), 1510 (Ar) cm^-1^. C_28_H_22_O_4_Br_2_; Calcd. C, 57.76; H, 3.81 found C, 57.81; H, 4.01. m.p. = 239.0–245.0 K

### Refinement

All H atoms were found in a difference map and were subsequently refined as riding atoms, with C—H = 0.95 (aromatic) and 0.98 (methyl) Å, and with *U*_ĩso_(H) = 1.2 *U*_eq_(C).

### Crystal data

**Table:**

C_28_H_22_Br_2_O_4_	*F*(000) = 1168
*M**_r_* = 582.28	*D*_x_ = 1.626 Mg m^−^^3^
Monoclinic, *P*2_1_/*n*	Melting point = 239.0–245.0 K
Hall symbol: -P 2yn	Cu *K*α radiation, λ = 1.54187 Å
*a* = 11.5948 (2) Å	Cell parameters from 30055 reflections
*b* = 8.37239 (15) Å	θ = 3.6–68.2°
*c* = 24.5352 (5) Å	µ = 4.60 mm^−^^1^
β = 92.617 (1)°	*T* = 193 K
*V* = 2379.29 (8) Å^3^	Block, yellow
*Z* = 4	0.50 × 0.40 × 0.20 mm

### Data collection

**Table:**

Rigaku R-AXIS RAPID diffractometer	4319 independent reflections
Radiation source: rotating anode	3916 reflections with *I* > 2σ(*I*)
graphite	*R*_int_ = 0.079
Detector resolution: 10.000 pixels mm^-1^	θ_max_ = 68.2°, θ_min_ = 3.6°
ω scans	*h* = −13→13
Absorption correction: numerical (*NUMABS*; Higashi, 1999)	*k* = −10→10
*T*_min_ = 0.207, *T*_max_ = 0.460	*l* = −29→29
41450 measured reflections	

### Refinement

**Table:**

Refinement on *F*^2^	Secondary atom site location: difference Fourier map
Least-squares matrix: full	Hydrogen site location: inferred from neighbouring sites
*R*[*F*^2^ > 2σ(*F*^2^)] = 0.041	H-atom parameters constrained
*wR*(*F*^2^) = 0.103	*w* = 1/[σ^2^(*F*_o_^2^) + (0.0277*P*)^2^ + 4.5808*P*] where *P* = (*F*_o_^2^ + 2*F*_c_^2^)/3
*S* = 1.14	(Δ/σ)_max_ < 0.001
4319 reflections	Δρ_max_ = 0.85 e Å^−^^3^
310 parameters	Δρ_min_ = −0.59 e Å^−^^3^
0 restraints	Extinction correction: *SHELXL97* (Sheldrick, 2008), Fc^*^=kFc[1+0.001xFc^2^λ^3^/sin(2θ)]^-1/4^
Primary atom site location: structure-invariant direct methods	Extinction coefficient: 0.00183 (10)

### Special details

**Table:**

Geometry. All e.s.d.'s (except the e.s.d. in the dihedral angle between two l.s. planes) are estimated using the full covariance matrix. The cell e.s.d.'s are taken into account individually in the estimation of e.s.d.'s in distances, angles and torsion angles; correlations between e.s.d.'s in cell parameters are only used when they are defined by crystal symmetry. An approximate (isotropic) treatment of cell e.s.d.'s is used for estimating e.s.d.'s involving l.s. planes.
Refinement. Refinement of *F*^2^ against ALL reflections. The weighted *R*-factor *wR* and goodness of fit *S* are based on *F*^2^, conventional *R*-factors *R* are based on *F*, with *F* set to zero for negative *F*^2^. The threshold expression of *F*^2^ > σ(*F*^2^) is used only for calculating *R*-factors(gt) *etc*. and is not relevant to the choice of reflections for refinement. *R*-factors based on *F*^2^ are statistically about twice as large as those based on *F*, and *R*- factors based on ALL data will be even larger.

### Fractional atomic coordinates and isotropic or equivalent isotropic displacement parameters (Å^2^)

**Table:**

	*x*	*y*	*z*	*U*_iso_*/*U*_eq_	
Br1	−0.15947 (4)	0.20415 (5)	0.241294 (16)	0.04354 (16)	
Br2	−0.11445 (4)	0.28738 (5)	−0.255555 (15)	0.04200 (15)	
O1	0.1773 (2)	0.4762 (3)	0.01077 (9)	0.0310 (5)	
O2	0.1901 (2)	0.0544 (3)	0.00410 (9)	0.0317 (5)	
O3	0.33170 (19)	0.5296 (3)	0.13097 (9)	0.0331 (6)	
O4	0.3673 (2)	0.0170 (3)	−0.10273 (9)	0.0352 (6)	
C1	0.3426 (3)	0.3654 (4)	0.05473 (12)	0.0229 (6)	
C2	0.3998 (3)	0.4487 (4)	0.09594 (12)	0.0268 (7)	
C3	0.5212 (3)	0.4562 (4)	0.10035 (13)	0.0300 (7)	
H3	0.5592	0.5116	0.1298	0.036*	
C4	0.5834 (3)	0.3832 (4)	0.06193 (13)	0.0296 (7)	
H4	0.6651	0.3932	0.0637	0.035*	
C5	0.5291 (3)	0.2925 (4)	0.01916 (13)	0.0262 (7)	
C6	0.5961 (3)	0.2128 (4)	−0.01911 (14)	0.0301 (7)	
H6	0.6779	0.2216	−0.0163	0.036*	
C7	0.5455 (3)	0.1239 (4)	−0.05987 (14)	0.0312 (7)	
H7	0.5916	0.0719	−0.0855	0.037*	
C8	0.4242 (3)	0.1090 (4)	−0.06393 (13)	0.0277 (7)	
C9	0.3552 (3)	0.1836 (4)	−0.02723 (12)	0.0234 (7)	
C10	0.4066 (3)	0.2799 (4)	0.01571 (12)	0.0230 (6)	
C11	0.2132 (3)	0.3889 (4)	0.04739 (12)	0.0234 (6)	
C12	0.1329 (3)	0.3064 (4)	0.08379 (12)	0.0230 (6)	
C13	0.1743 (3)	0.2117 (4)	0.12720 (12)	0.0256 (7)	
H13	0.2551	0.1999	0.1342	0.031*	
C14	0.0980 (3)	0.1351 (4)	0.15989 (13)	0.0294 (7)	
H14	0.1267	0.0730	0.1899	0.035*	
C15	−0.0205 (3)	0.1477 (4)	0.14942 (13)	0.0280 (7)	
C16	−0.0608 (3)	0.2401 (4)	0.10524 (14)	0.0310 (7)	
H16	−0.1416	0.2481	0.0972	0.037*	
C17	0.0147 (3)	0.3201 (4)	0.07308 (13)	0.0269 (7)	
H17	−0.0141	0.3844	0.0436	0.032*	
C18	0.2281 (3)	0.1422 (4)	−0.03013 (12)	0.0244 (7)	
C19	0.1506 (3)	0.2140 (4)	−0.07415 (12)	0.0247 (7)	
C20	0.1954 (3)	0.3069 (4)	−0.11521 (13)	0.0264 (7)	
H20	0.2764	0.3231	−0.1161	0.032*	
C21	0.1222 (3)	0.3757 (4)	−0.15465 (13)	0.0282 (7)	
H21	0.1534	0.4372	−0.1830	0.034*	
C22	0.0038 (3)	0.3556 (4)	−0.15305 (13)	0.0269 (7)	
C23	−0.0412 (3)	0.2637 (4)	−0.11166 (14)	0.0303 (7)	
H23	−0.1223	0.2499	−0.1103	0.036*	
C24	0.0318 (3)	0.1926 (4)	−0.07264 (13)	0.0290 (7)	
H24	0.0007	0.1290	−0.0448	0.035*	
C25	0.3876 (3)	0.6214 (5)	0.17364 (14)	0.0388 (9)	
H25A	0.4311	0.5499	0.1986	0.047*	
H25B	0.3294	0.6790	0.1937	0.047*	
H25C	0.4404	0.6981	0.1579	0.047*	
C26	0.4333 (3)	−0.0513 (5)	−0.14481 (14)	0.0388 (9)	
H26A	0.4684	0.0343	−0.1657	0.047*	
H26B	0.3825	−0.1154	−0.1692	0.047*	
H26C	0.4941	−0.1195	−0.1283	0.047*	
C27	−0.1034 (3)	0.0618 (4)	0.18381 (15)	0.0362 (8)	
H27A	−0.0648	−0.0323	0.2009	0.043*	
H27B	−0.1697	0.0235	0.1605	0.043*	
C28	−0.0739 (3)	0.4356 (4)	−0.19505 (14)	0.0346 (8)	
H28A	−0.0348	0.5307	−0.2095	0.041*	
H28B	−0.1452	0.4717	−0.1780	0.041*	

### Atomic displacement parameters (Å^2^)

**Table:**

	*U*^11^	*U*^22^	*U*^33^	*U*^12^	*U*^13^	*U*^23^
Br1	0.0452 (3)	0.0507 (3)	0.0359 (2)	−0.00291 (18)	0.01501 (18)	−0.00418 (17)
Br2	0.0488 (3)	0.0514 (3)	0.0252 (2)	0.00179 (18)	−0.00500 (16)	−0.00459 (16)
O1	0.0332 (13)	0.0350 (13)	0.0247 (11)	0.0049 (10)	−0.0006 (9)	0.0078 (10)
O2	0.0350 (13)	0.0305 (12)	0.0299 (12)	−0.0011 (10)	0.0063 (10)	0.0043 (10)
O3	0.0292 (12)	0.0422 (14)	0.0282 (12)	−0.0060 (11)	0.0036 (10)	−0.0113 (10)
O4	0.0355 (13)	0.0374 (14)	0.0330 (13)	0.0077 (11)	0.0051 (10)	−0.0102 (10)
C1	0.0228 (16)	0.0245 (16)	0.0212 (15)	−0.0007 (13)	0.0016 (12)	0.0044 (12)
C2	0.0308 (17)	0.0280 (16)	0.0217 (15)	−0.0033 (14)	0.0027 (13)	0.0043 (13)
C3	0.0293 (18)	0.0342 (18)	0.0260 (16)	−0.0056 (14)	−0.0037 (14)	0.0040 (14)
C4	0.0224 (16)	0.0318 (18)	0.0342 (18)	−0.0024 (14)	−0.0021 (14)	0.0111 (14)
C5	0.0260 (17)	0.0268 (16)	0.0260 (16)	0.0040 (13)	0.0017 (13)	0.0100 (13)
C6	0.0246 (17)	0.0317 (18)	0.0344 (18)	0.0068 (14)	0.0048 (14)	0.0100 (14)
C7	0.0321 (18)	0.0313 (18)	0.0310 (17)	0.0094 (15)	0.0091 (14)	0.0083 (14)
C8	0.0334 (18)	0.0258 (16)	0.0242 (16)	0.0053 (14)	0.0021 (13)	0.0057 (13)
C9	0.0255 (16)	0.0217 (15)	0.0231 (15)	0.0039 (12)	0.0025 (13)	0.0052 (12)
C10	0.0236 (16)	0.0227 (15)	0.0225 (15)	0.0009 (12)	0.0000 (12)	0.0073 (12)
C11	0.0271 (16)	0.0233 (15)	0.0197 (15)	0.0028 (13)	−0.0014 (12)	−0.0040 (12)
C12	0.0258 (16)	0.0225 (15)	0.0208 (15)	0.0015 (12)	0.0028 (12)	−0.0040 (12)
C13	0.0227 (16)	0.0304 (17)	0.0237 (16)	0.0000 (13)	−0.0009 (13)	−0.0007 (13)
C14	0.0346 (19)	0.0290 (17)	0.0245 (16)	−0.0020 (14)	0.0003 (14)	0.0006 (13)
C15	0.0312 (18)	0.0248 (16)	0.0287 (17)	−0.0027 (14)	0.0078 (14)	−0.0078 (13)
C16	0.0235 (17)	0.0353 (18)	0.0343 (18)	0.0014 (14)	0.0023 (14)	−0.0061 (14)
C17	0.0247 (17)	0.0308 (17)	0.0252 (16)	0.0026 (13)	−0.0002 (13)	−0.0016 (13)
C18	0.0292 (17)	0.0212 (15)	0.0230 (15)	0.0005 (13)	0.0031 (13)	−0.0034 (12)
C19	0.0292 (17)	0.0242 (16)	0.0208 (15)	0.0007 (13)	0.0007 (13)	−0.0050 (12)
C20	0.0246 (17)	0.0265 (16)	0.0282 (16)	0.0004 (13)	0.0027 (13)	−0.0023 (13)
C21	0.0351 (19)	0.0256 (16)	0.0241 (16)	−0.0001 (14)	0.0018 (13)	−0.0017 (13)
C22	0.0304 (17)	0.0236 (16)	0.0264 (16)	0.0021 (13)	−0.0012 (13)	−0.0077 (13)
C23	0.0236 (17)	0.0351 (19)	0.0322 (18)	0.0005 (14)	0.0021 (14)	−0.0067 (14)
C24	0.0294 (18)	0.0326 (18)	0.0252 (16)	−0.0026 (14)	0.0040 (14)	−0.0028 (13)
C25	0.044 (2)	0.047 (2)	0.0257 (17)	−0.0160 (18)	0.0029 (15)	−0.0096 (16)
C26	0.052 (2)	0.0338 (19)	0.0317 (19)	0.0043 (17)	0.0130 (16)	−0.0044 (15)
C27	0.038 (2)	0.0310 (18)	0.041 (2)	−0.0066 (15)	0.0120 (16)	−0.0063 (15)
C28	0.0372 (19)	0.0322 (18)	0.0336 (18)	0.0039 (15)	−0.0053 (15)	−0.0060 (15)

### Geometric parameters (Å, °)

**Table:**

Br1—C27	1.979 (3)	C14—C15	1.390 (5)
Br2—C28	1.975 (3)	C14—H14	0.9500
O1—C11	1.216 (4)	C15—C16	1.395 (5)
O2—C18	1.214 (4)	C15—C27	1.492 (5)
O3—C2	1.372 (4)	C16—C17	1.379 (5)
O3—C25	1.430 (4)	C16—H16	0.9500
O4—C8	1.370 (4)	C17—H17	0.9500
O4—C26	1.432 (4)	C18—C19	1.499 (4)
C1—C2	1.374 (4)	C19—C20	1.392 (5)
C1—C10	1.430 (4)	C19—C24	1.392 (5)
C1—C11	1.516 (4)	C20—C21	1.383 (4)
C2—C3	1.409 (5)	C20—H20	0.9500
C3—C4	1.357 (5)	C21—C22	1.386 (5)
C3—H3	0.9500	C21—H21	0.9500
C4—C5	1.419 (5)	C22—C23	1.395 (5)
C4—H4	0.9500	C22—C28	1.496 (4)
C5—C6	1.413 (5)	C23—C24	1.383 (5)
C5—C10	1.423 (4)	C23—H23	0.9500
C6—C7	1.358 (5)	C24—H24	0.9500
C6—H6	0.9500	C25—H25A	0.9800
C7—C8	1.411 (5)	C25—H25B	0.9800
C7—H7	0.9500	C25—H25C	0.9800
C8—C9	1.380 (4)	C26—H26A	0.9800
C9—C10	1.434 (4)	C26—H26B	0.9800
C9—C18	1.513 (4)	C26—H26C	0.9800
C11—C12	1.489 (4)	C27—H27A	0.9900
C12—C17	1.388 (4)	C27—H27B	0.9900
C12—C13	1.396 (4)	C28—H28A	0.9900
C13—C14	1.379 (5)	C28—H28B	0.9900
C13—H13	0.9500		
			
C2—O3—C25	118.0 (3)	C15—C16—H16	119.5
C8—O4—C26	118.0 (3)	C16—C17—C12	119.9 (3)
C2—C1—C10	119.9 (3)	C16—C17—H17	120.0
C2—C1—C11	117.6 (3)	C12—C17—H17	120.0
C10—C1—C11	121.8 (3)	O2—C18—C19	121.1 (3)
O3—C2—C1	116.1 (3)	O2—C18—C9	119.3 (3)
O3—C2—C3	122.1 (3)	C19—C18—C9	119.5 (3)
C1—C2—C3	121.8 (3)	C20—C19—C24	119.6 (3)
C4—C3—C2	119.1 (3)	C20—C19—C18	121.0 (3)
C4—C3—H3	120.4	C24—C19—C18	119.4 (3)
C2—C3—H3	120.4	C21—C20—C19	120.1 (3)
C3—C4—C5	121.6 (3)	C21—C20—H20	119.9
C3—C4—H4	119.2	C19—C20—H20	119.9
C5—C4—H4	119.2	C20—C21—C22	120.4 (3)
C6—C5—C4	120.4 (3)	C20—C21—H21	119.8
C6—C5—C10	120.2 (3)	C22—C21—H21	119.8
C4—C5—C10	119.4 (3)	C21—C22—C23	119.5 (3)
C7—C6—C5	121.1 (3)	C21—C22—C28	119.5 (3)
C7—C6—H6	119.5	C23—C22—C28	121.0 (3)
C5—C6—H6	119.5	C24—C23—C22	120.3 (3)
C6—C7—C8	119.8 (3)	C24—C23—H23	119.9
C6—C7—H7	120.1	C22—C23—H23	119.9
C8—C7—H7	120.1	C23—C24—C19	120.1 (3)
O4—C8—C9	115.7 (3)	C23—C24—H24	120.0
O4—C8—C7	123.0 (3)	C19—C24—H24	120.0
C9—C8—C7	121.3 (3)	O3—C25—H25A	109.5
C8—C9—C10	120.0 (3)	O3—C25—H25B	109.5
C8—C9—C18	117.2 (3)	H25A—C25—H25B	109.5
C10—C9—C18	122.3 (3)	O3—C25—H25C	109.5
C5—C10—C1	118.1 (3)	H25A—C25—H25C	109.5
C5—C10—C9	117.7 (3)	H25B—C25—H25C	109.5
C1—C10—C9	124.2 (3)	O4—C26—H26A	109.5
O1—C11—C12	121.3 (3)	O4—C26—H26B	109.5
O1—C11—C1	118.0 (3)	H26A—C26—H26B	109.5
C12—C11—C1	120.7 (3)	O4—C26—H26C	109.5
C17—C12—C13	119.6 (3)	H26A—C26—H26C	109.5
C17—C12—C11	119.1 (3)	H26B—C26—H26C	109.5
C13—C12—C11	121.3 (3)	C15—C27—Br1	110.7 (2)
C14—C13—C12	120.0 (3)	C15—C27—H27A	109.5
C14—C13—H13	120.0	Br1—C27—H27A	109.5
C12—C13—H13	120.0	C15—C27—H27B	109.5
C13—C14—C15	120.8 (3)	Br1—C27—H27B	109.5
C13—C14—H14	119.6	H27A—C27—H27B	108.1
C15—C14—H14	119.6	C22—C28—Br2	110.6 (2)
C14—C15—C16	118.6 (3)	C22—C28—H28A	109.5
C14—C15—C27	121.0 (3)	Br2—C28—H28A	109.5
C16—C15—C27	120.3 (3)	C22—C28—H28B	109.5
C17—C16—C15	121.0 (3)	Br2—C28—H28B	109.5
C17—C16—H16	119.5	H28A—C28—H28B	108.1
			
C25—O3—C2—C1	−178.4 (3)	C10—C1—C11—C12	110.7 (3)
C25—O3—C2—C3	−1.2 (5)	O1—C11—C12—C17	4.9 (4)
C10—C1—C2—O3	178.9 (3)	C1—C11—C12—C17	−174.7 (3)
C11—C1—C2—O3	8.1 (4)	O1—C11—C12—C13	−177.3 (3)
C10—C1—C2—C3	1.6 (5)	C1—C11—C12—C13	3.1 (4)
C11—C1—C2—C3	−169.1 (3)	C17—C12—C13—C14	−1.4 (5)
O3—C2—C3—C4	−175.0 (3)	C11—C12—C13—C14	−179.2 (3)
C1—C2—C3—C4	2.1 (5)	C12—C13—C14—C15	1.6 (5)
C2—C3—C4—C5	−3.5 (5)	C13—C14—C15—C16	−0.3 (5)
C3—C4—C5—C6	−177.6 (3)	C13—C14—C15—C27	178.4 (3)
C3—C4—C5—C10	1.2 (5)	C14—C15—C16—C17	−1.2 (5)
C4—C5—C6—C7	179.2 (3)	C27—C15—C16—C17	−180.0 (3)
C10—C5—C6—C7	0.4 (5)	C15—C16—C17—C12	1.4 (5)
C5—C6—C7—C8	−0.7 (5)	C13—C12—C17—C16	−0.1 (5)
C26—O4—C8—C9	174.4 (3)	C11—C12—C17—C16	177.8 (3)
C26—O4—C8—C7	−7.7 (4)	C8—C9—C18—O2	104.4 (3)
C6—C7—C8—O4	−177.7 (3)	C10—C9—C18—O2	−67.8 (4)
C6—C7—C8—C9	0.1 (5)	C8—C9—C18—C19	−77.4 (4)
O4—C8—C9—C10	178.7 (3)	C10—C9—C18—C19	110.3 (3)
C7—C8—C9—C10	0.7 (5)	O2—C18—C19—C20	−176.1 (3)
O4—C8—C9—C18	6.3 (4)	C9—C18—C19—C20	5.7 (4)
C7—C8—C9—C18	−171.7 (3)	O2—C18—C19—C24	6.4 (5)
C6—C5—C10—C1	−178.7 (3)	C9—C18—C19—C24	−171.7 (3)
C4—C5—C10—C1	2.4 (4)	C24—C19—C20—C21	−0.8 (5)
C6—C5—C10—C9	0.5 (4)	C18—C19—C20—C21	−178.3 (3)
C4—C5—C10—C9	−178.4 (3)	C19—C20—C21—C22	1.3 (5)
C2—C1—C10—C5	−3.8 (4)	C20—C21—C22—C23	−0.8 (5)
C11—C1—C10—C5	166.6 (3)	C20—C21—C22—C28	178.0 (3)
C2—C1—C10—C9	177.0 (3)	C21—C22—C23—C24	−0.3 (5)
C11—C1—C10—C9	−12.6 (5)	C28—C22—C23—C24	−179.0 (3)
C8—C9—C10—C5	−1.0 (4)	C22—C23—C24—C19	0.8 (5)
C18—C9—C10—C5	171.0 (3)	C20—C19—C24—C23	−0.2 (5)
C8—C9—C10—C1	178.2 (3)	C18—C19—C24—C23	177.3 (3)
C18—C9—C10—C1	−9.8 (5)	C14—C15—C27—Br1	96.1 (3)
C2—C1—C11—O1	101.7 (3)	C16—C15—C27—Br1	−85.1 (3)
C10—C1—C11—O1	−68.9 (4)	C21—C22—C28—Br2	95.9 (3)
C2—C1—C11—C12	−78.7 (4)	C23—C22—C28—Br2	−85.4 (3)

### Hydrogen-bond geometry (Å, °)

**Table:**

*D*—H···*A*	*D*—H	H···*A*	*D*···*A*	*D*—H···*A*
C17—H17···O1^i^	0.95	2.55	3.417 (4)	152
C28—H28B···O3^i^	0.99	2.50	3.453 (4)	162

Symmetry codes: (i) −*x*, −*y*+1, −*z*.
